# Does daily wear time of Twin Block reliably predict its efficiency of class II treatment?

**DOI:** 10.1007/s00056-021-00300-7

**Published:** 2021-05-07

**Authors:** Michal Sarul, Marek Nahajowski, Grzegorz Gawin, Joanna Antoszewska-Smith

**Affiliations:** Department of Orthodontics and Dentofacial Orthopedics, Wroclaw Medical University, Krakowska 26, 50-425 Wroclaw, Poland

**Keywords:** Functional treatment, Angle class II malocclusion, Overjet, Treatment adherence and compliance, Microsensors, Funktionelle Behandlung, Angle-Klasse-II-Malokklusion, Overjet, Behandlungsadhärenz und Compliance, Mikrosensoren

## Abstract

**Purpose:**

The objective of this study was to investigate how daily wear time (DWT) influences class II malocclusion treatment efficiency.

**Materials and methods:**

The study group consisted of 55 patients (mean age 10.4 years) diagnosed with a class II/1 malocclusion. Twin block appliances, with built-in Theramon® microsensors (MC Technology, Hargelsberg, Austria) to monitor patients’ cooperation (daily wear time assessment), were used for treatment. Cephalograms were taken and the following initial and final measurements were compared: Co-Gn, Co-Go, Co-Olp, Pg-Olp, WITS, SNA, SNB, ANB, Co-Go-Me, overjet, molar and canine relationships. The Shapiro–Wilk test, Wilcoxon signed-rank test, Student’s t-test, Levene’s test, Mann–Whitney U test, Kruskal–Wallis test, χ^2^ test, and Spearman’s rank correlation coefficient with *p* < 0.05 set as the statistical significance level were used to determine the correlation of the outcomes with DWT; a ROC (receiver operating characteristic) curve was calculated to illustrate diagnostic ability of the binary classifier system.

**Results:**

DWT was very highly positively correlated with change of the Pg-Olp parameter and highly with an improvement in the ANB, SNA, and SNB angles, an increase in the WITS parameter and an increase in Co-Gn distance. DWTs < 7.5 h correlated with significantly less improvement of the investigated variables. However, DWT > 7.5 h did not significantly correlate with the improvement of the overjet and most of the linear parameters in the mandible. The ROC curve and its AUC (area under curve) allowed the determination of a DWT of 7 h and 48 min to be capable of establishing a class I relationship with 83% probability.

**Conclusions:**

Class II treatment efficiency was influenced by DWT; an 8 h threshold value had an 83% probability of establishing a class I relationship.

## Abstract (de)

**Zielsetzung:**

Ziel dieser Studie war es zu untersuchen, wie die tägliche Tragezeit (DWT) die Effizienz der Behandlung von Klasse-II-Malokklusionen beeinflusst.

**Materialien und Methoden:**

Die Studiengruppe bestand aus 55 Patienten (Durchschnittsalter 10,4 Jahre), bei denen eine Klasse-II/1-Malokklusion diagnostiziert wurde. Für die Behandlung wurden Twin-Block-Apparaturen mit eingebauten Theramon®-Mikrosensoren (MC Technology, Hargelsberg, Österreich) zur Überwachung der Mitarbeit der Patienten (tägliche Tragezeitbewertung) verwendet. Es wurden Kephalogramme aufgenommen und die folgenden Anfangs- und Endmessungen verglichen: Co-Gn, Co-Go, Co-Olp, Pg-Olp, WITS, SNA, SNB, ANB, Co-Go-Me, Overjet, Molaren- und Eckzahnbeziehungen. Der Shapiro-Wilk-Test, der Wilcoxon-Vorzeichen-Rang-Test, der Student-t-Test, der Levene-Test, der Mann-Whitney-U-Test, der Kruskal-Wallis-Test, der χ^2^-Test und der Rangkorrelationskoeffizient nach Spearman mit *p* < 0.05 als statistisches Signifikanzniveau wurden verwendet, um die Korrelation der Ergebnisse mit der DWT zu bestimmen; eine ROC(„receiver operating characteristic“)-Kurve wurde berechnet, um die diagnostische Fähigkeit des binären Klassifizierungssystems zu veranschaulichen.

**Ergebnisse:**

Die DWT korrelierte sehr stark positiv mit der Veränderung des Pg-Olp-Parameters, hoch mit einer Verbesserung der ANB-, SNA- und SNB-Winkel, mit einer Erhöhung des WITS-Parameters und mit einer Erhöhung des Co-Gn-Abstands. DWTs < 7.5 h korrelierten mit deutlich weniger Verbesserung der untersuchten Variablen. DWTs > 7.5 h korrelierten jedoch nicht signifikant mit der Verbesserung des Overjet und der meisten linearen Parameter im Unterkiefer. Mithilfe der ROC-Kurve und ihrer AUC („area under curve“) konnte bei einer DWT von 7 h und 48 min mit 83%iger Wahrscheinlichkeit ein Zusammenhang der Klasse I festgestellt werden.

**Schlussfolgerungen:**

Die Effizienz der Klasse-II-Behandlung wurde durch die DWT beeinflusst; ein Schwellenwert von 8 h war mit 83%iger Wahrscheinlichkeit in der Lage, eine Klasse-I-Beziehung zu etablieren.

## Introduction

Class II malocclusion is one of the most common abnormalities, occurring in approximately 19% of the world population. In patients belonging to the Caucasian race, it is estimated that a class II malocclusion is present in up to 23% in permanent dentition and 26% in mixed dentition [2, 14, 28]. In cases where the etiology of malocclusion lies in abnormal maxillary and/or mandibular growth, its direction and rate may be modified by functional treatment [5, 12, 24]. Unfortunately, the effectiveness of such treatment is still debatable, and the results of studies examining this issue are often contradictory [7, 10, 15–17, 19, 21, 29, 31]. Regardless of this controversy, many authors believe that one of the factors responsible for improving the skeletal pattern is the daily wear time (DWT) of removable appliances [18, 26]. Until recently, there were two major drawbacks:

- No objective methods have been described to control DWT.
- Its value recommended for treating a malocclusion was determined based on observations rather than evidence-based research.

The Theramon® System (MC Technology, Hargelsberg, Austria) overcame the first difficulty. This system consists of temperature-sensitive microsensors built into the appliance, having no effect on sensations when individuals are wearing the appliance; these appliances are also resistant to manipulation by the patient, making it possible to identify such behavior and estimate the wear time with an accuracy of 15 min [27]. Microsensors have provided evidence that patients wear their functional appliances for a shorter time than the recommended 12–14 h a day [1, 25, 26]. Nevertheless, the question of what DWT threshold value allows for effective functional treatment remains unsettled. This is particularly important in terms of common malocclusion treatment, which is paid for by public funds provided that removable appliances are used, despite their theoretically lower effectiveness. Therefore, this study aims to objectively determine whether and to what extent the functional treatment of class II malocclusion with removable appliances depends on the DWT and whether there is a threshold value of the DWT required for this treatment to be effective.

## Materials and methods

Prior to beginning the investigation, the study obtained Bioethics Committee approval No. KB-322/2014 (Bioethics Committee of Wroclaw Medical University, Poland).

Inclusion criteria for the study were

- Mixed dentition,
- Cervical vertebral maturation at the CVMS2 (CVMS: cervical vertebral maturation stage),
- Mild or moderate skeletal class II malocclusions (4.5° ≤ ANB ≤ 8.0°),
- Full dental class II,
- Lack of features of an open bite (ML/NL = 20.0° ± 7.0°),
- No history of prior orthodontic treatment.

Exclusion criteria for the study were

- Lack of consent to participate in the study or to be treated with a removable appliance,
- Cervical vertebral maturation at a stage higher than CVMS2,
- Congenital disabilities of the craniofacial region,
- Contraindications to functional treatment—protrusion of the mandibular incisors (ML:L1 > 101°).

**Fig. 1:**
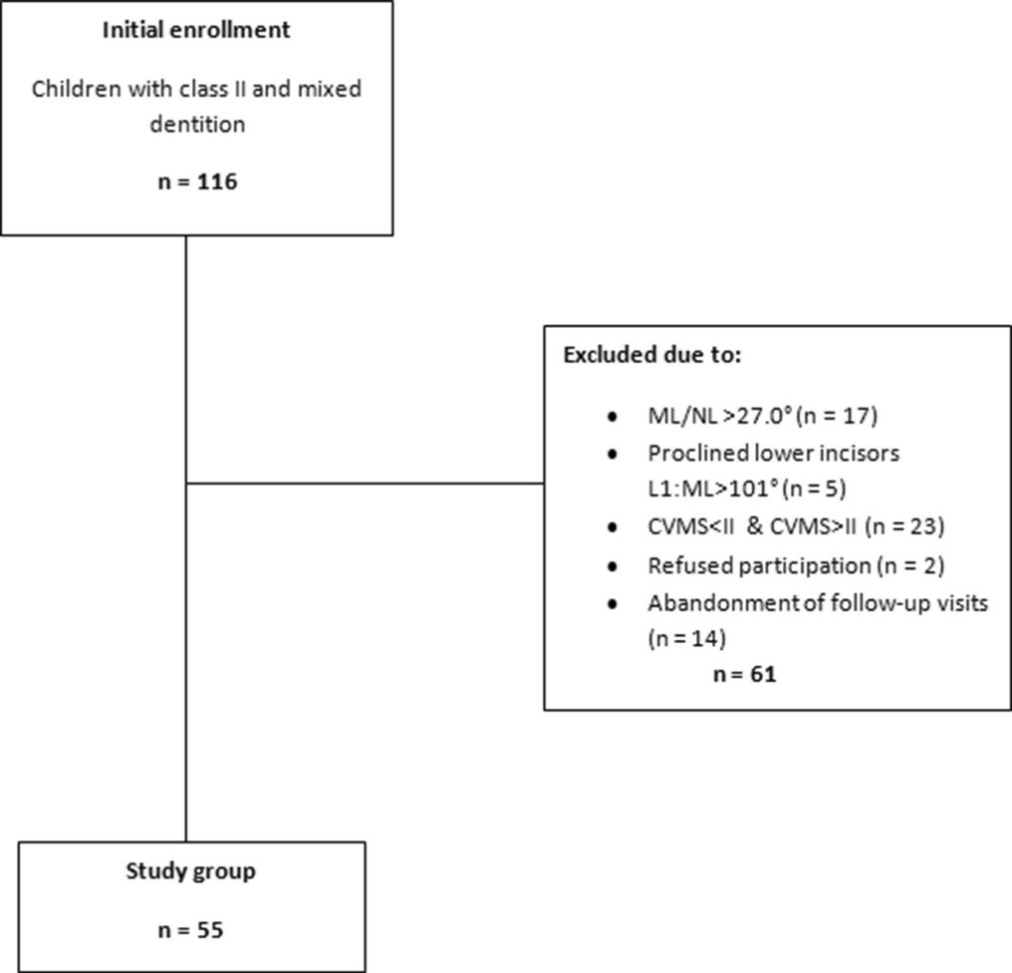
Flowchart of the study material allocation Flussdiagramm zur Verteilung des in der Studie verwendeten Materials

Out of 116 individuals, we excluded 47 children. Thus, 69 patients qualified for treatment with the Twin Block appliance (Fig. 1). All patients and their parents/guardians were informed about the objective of the study. All parents/guardians signed an informed assent form for their children’s participation in the study.

**Fig. 2:**
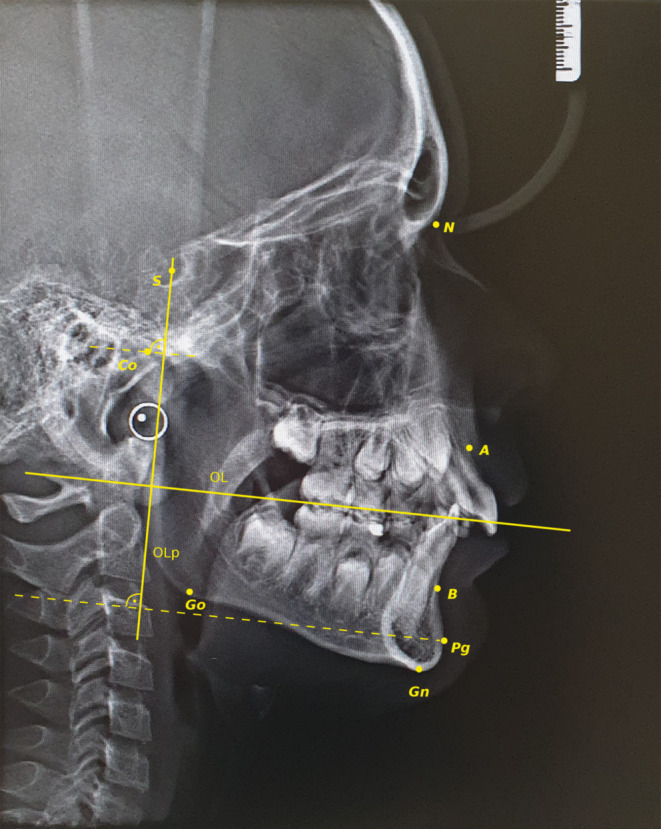
Cephalometric points assessed in the study. *S* Sella, *N* Nasion, *A* Subspinale, *B* Supramentale, *Pg* Pogonion, *Gn* Gnation, *Go* Gonion, *Co* Condylion, *OL* functional occlusal line, *OLp* line drawn through Sella, which is perpendicular to occlusal line, *Co-Olp* distance between Condylion and OLp line, *Pg-Olp* distance between Pogonion and OLp line Für die Studie ermittelte kephalometrische Punkte: *S* Sella, *N* Nasion, *A* Subspinale, *B* Supramentale, *Pg* Pogonion, *Gn* Gnation, *Go* Gonion, *Co* Condylion, *OL* funktionelle Okklusionsebene, *OLp* Linie durch den Punkt Sella, senkrecht zur Okklusionsebene, *Co-Olp* Abstand zwischen Condylion und OLp-Linie, *Pg-Olp* Abstand zwischen Pogonion und OLp-Linie

A single clinician evaluated the patients’ CVMS based on the initial lateral cephalograms. At the beginning of treatment (T1), overjet and the following values of cephalometric parameters (Fig. 2) were registered:

- SNA (°)
- SNB (°)
- ANB (°)
- Co-Go-Me (°)
- WITS (mm)
- Co-Gn (mm)
- Co-Go (mm)
- Co-Olp (mm)
- Pg-Olp (mm)

All patients received conventional Twin Block appliances with passive labial arches. The construction bite secured forward posturing of the mandible to achieve a class I molar relationship, as well as vertical disorientation of 4–6 mm measured between the molars. Every appliance was equipped with a built-in Theramon® microsensor. The patients and their parents/guardians were instructed that the appliance should be worn at least 8–10 h at night and 2–4 h during the day, i.e., 12–14 h per day. During follow-up visits, every 4–6 weeks, the data collected by each sensor were read by a Theramon® reader connected to a personal computer via a USB device. Treatment lasted 18 months (±1 month). At that moment (T2), the mean value of the DWT was calculated for each patient, as were both the overjet and the cephalometric parameters posttreatment. The clinician took all measurements twice, at a 2-week interval, with the mean of both values being analyzed. The presence or lack of class I canine and molar relationships were recorded for all patients. A total of 14 patients missed their follow-up visits. Eventually, the study group comprised 55 Caucasian patients, 26 boys and 29 girls aged 9.4–11.4 years (mean 10.4 years), whose records underwent statistical analysis.

Because nontreatment of patients with a high index of orthodontic treatment need (IOTN) value seemed to be prohibited by ethical reasons, this study used control groups reported in other papers [3, 4, 6, 9], with similar eligibility criteria (Table 1).

**Table 1:** Characteristics of the control groups Charakteristika der Kontrollgruppen

Baccetti et al. [3]	Ghislanzoni et al. [9]	Cozza et al. [6]	Baysal and Uysal [4]
*n* = 14	*n* = 17	*n* = 30	*n* = 20
Class II CVS III	ANB ≥ 4° Angle class II CVMS II	Overjet > 5 mm Angle class II ANB > 5° SNB < 78°	ANB > 4° SNB < 78° Overjet ≥ 5 mm SN-GoGn = 32° ± 6° Angle class II Patients with fourth (S and H2) or fifth (MP3cap, PP1cap, Rcap) epiphyseal stages on hand–wrist radiograph
7 girls and 7 boys	Female and male sex	15 girls and 15 boys	9 girls and 11 boys
Mean age: 13 years 7 months	–	9–11 years (mean age: 10)	Mean age: 12.17 years

*CVMS* cervical vertebral maturation stage

### Statistical analysis

The calculations were performed in Statistica for Windows 10. The following statistical tests were used to compare data from T1 and T2 periods: Shapiro–Wilk test, Wilcoxon signed-rank test, Student’s t-test, Levene’s test, Mann–Whitney U test, Kruskal–Wallis test, χ^2^ test, and Spearman’s rank correlation coefficient; *p* < 0.05 indicated the statistical significance level. Finally, a ROC (receiver operating characteristic) curve was drawn.

## Results

The mean daily wear time was 7.60 ± 3.12 h/24 h. Statistics describing the DWT and range of changes obtained in the period from T1 to T2 are presented in Table 2. The mean values of overjet and all cephalometric parameters changed significantly (*p* < 0.05; Table 3). Namely, there was a reduction in overjet and WITS parameter. A decrease in SNA and ANB angles and an increase in all linear measurements and in the SNB angle were observed.

**Table 2:** Descriptive statistics of the variables Deskriptive Statistik der Variablen

Variable	T1				T2			
	Min	Max	Mean	SD	Min	Max	Mean	SD
Overjet	6.70	10.30	8.19	0.93	2.80	14.00	7.01	3.26
Co-Gn (mm)	107.20	112.40	109.39	1.21	111.00	119.30	114.42	1.90
Co-Go (mm)	55.40	59.10	57.15	0.94	57.00	62.40	59.78	1.13
Co-Olp	9.60	14.90	11.69	1.24	8.50	19.10	12.67	2.47
Pg-Olp	74.80	90.80	82.75	4.70	75.80	95.90	86.63	5.02
WITS appraisal (mm)	4.40	6.80	5.35	0.54	−0.40	7.80	4.13	2.25
SNA (°)	77.20	81.10	79.62	0.87	76.10	81.40	79.27	1.05
SNB (°)	72.40	75.40	73.96	0.75	72.00	77.80	75.23	1.53
ANB (°)	4.20	6.90	5.68	0.60	0.60	7.40	4.06	1.91
Co-Go-Me (°)	122.00	129.00	125.31	1.70	121.10	131.00	125.85	2.27
DWT (h)	–				1.40	21.90	7.60	3.12

*SD* standard deviation, *min* minimum, *max* maximum, *DWT* daily wear time

**Table 3:** Statistical analysis of the variable changes achieved within T1–T2 period Statistische Analyse der erreichten Variablenänderungen innerhalb des Zeitraums T1–T2

Variable	Mean	SD	Test	*p*
Overjet	−1.18	3.28	W	0.016410
Co-Gn (mm)	5.03	1.61	S	0.000000
Co-Go (mm)	2.63	0.49	S	0.000000
Co-Olp	0.99	1.24	W	0.000001
Pg-Olp	3.89	1.81	W	0.000000
WITS appraisal (mm)	−1.21	2.24	S	0.000178
SNA (°)	−0.35	0.45	S	0.000001
SNB (°)	1.27	1.21	S	0.000000
ANB (°)	−1.62	1.61	W	0.000000
Co-Go-Me (°)	0.54	1.58	W	0.024900

*W* Wilcoxon test, *S* Student’s t‑test, *SD* standard deviation

A statistically significant correlation of all the examined variables with the DWT was demonstrated. This correlation was very high and positive for the parameter Pg-Olp. A longer DWT had a high correlation with an improvement in the angles ANB, SNA, SNB and with the WITS parameter; a longer DWT also demonstrated a high correlation with an increase in the Co-Gn distance and the Co-Go-Me angle. The parameters Co-Go and Co-Olp as well as overjet were moderately dependent on the DWT (Table 4).

**Table 4:** Analysis of correlation between the variable changes and the DWT Analyse der Korrelation zwischen Veränderungen der Variablen und DWT

Variable	Spearman’s correlation test		
	*n*	r	*p*
Overjet	55	−0.408014	0.001987
Co-Gn (mm)	55	0.736825	0.000000
Co-Go (mm)	55	0.556505	0.000010
Co-Olp	55	0.584264	0.000003
Pg-Olp	55	0.848208	0.000000
WITS appraisal (mm)	55	−0.694642	0.000000
SNA (°)	55	−0.706521	0.000000
SNB (°)	55	0.737044	0.000000
ANB (°)	55	−0.768877	0.000000
Co-Go-Me (°)	55	0.850272	0.000000

*DWT* daily wear time

The median DWT was 7.5 h. Table 5 shows the results of the comparative statistical analysis of changes in the values of overjet and cephalometric parameters from T1 to T2 in patients wearing the appliance for a shorter or longer time than the median DWT. A statistically significant, more considerable improvement of all continuous variables was demonstrated by patients adhering to a DWT > 7.5 compared to patients with a DWT < 7.5 h. The analysis did not indicate that a DWT longer than the median affected the improvement of the overjet and most of the linear changes obtained in the mandible. While a DWT longer than 7.5 h showed a weak correlation with an increase in the distances Co-Gn, Co-Go, and Co-Olp, shortening the DWT below the median demonstrated a moderate correlation with these changes. A similar, moderate correlation of a longer and shorter treatment time was found for the angular parameters, namely, SNA, SNB, and ANB. A DWT > 7.5 h strongly influenced the reduction in the WITS parameter (r = −0.77). In comparison, a significantly smaller increase in the distance Pg-Olp showed a strong correlation with a DWT < 7.5 h (r = 0.802).

**Table 5:** Statistical analysis of the variable changes in relation to the DWT < 7.5 h and the DWT > 7.5 h Statistische Analyse der Variablenänderungen in Abhängigkeit von DWT < 7,5 h und DWT > 7,5 h

Variable	Group	Descriptive statistics		Mann–Whitney U test	Spearman’s correlation test	
		Mean	SD	*p*	r	*p*
Overjet	DWT < 7.5 h	0.44	1.85	0.001941	0.016	0.934
	DWT > 7.5 h	−2.87	3.61		−0.097	0.629
Co-Gn	DWT < 7.5 h	3.97	1.33	0.000001	0.569	0.002
	DWT > 7.5 h	6.14	1.06		0.367	0.059
Co-Go	DWT < 7.5 h	2.44	0.54	0.000273	0.533	0.004
	DWT > 7.5 h	2.84	0.33		−0.048	0.812
Co-Olp	DWT < 7.5 h	0.31	0.71	0.000024	0.213	0.277
	DWT > 7.5 h	1.69	1.28		0.262	0.186
Pg -Olp	DWT < 7.5 h	2.60	1.10	0.000000	0.802	0.000
	DWT > 7.5 h	5.22	1.38		0.495	0.009
WITS appraisal	DWT < 7.5 h	0.24	1.27	0.000001	0.169	0.389
	DWT > 7.5 h	−2.72	2.02		−0.773	0.000002
SNA	DWT < 7.5 h	−0.07	0.32	0.000005	−0.541	0.003
	DWT > 7.5 h	−0.63	0.39		−0.421	0.028
SNB	DWT < 7.5 h	0.48	0.92	0.000001	0.445	0.018
	DWT > 7.5 h	2.08	0.89		0.474	0.013
ANB	DWT < 7.5 h	−0.55	1.10	0.000000	−0.461	0.013
	DWT > 7.5 h	−2.73	1.26		−0.495	0.009
Co-Go-Me	DWT < 7.5 h	−0.71	0.85	0.000000	–	–
	DWT > 7.5 h	1.83	1.01		–	–

*SD* standard deviation, *DWT* daily wear time

Changes of the parameters in the control groups were comparable with those achieved by the patients with a DWT < 7.5 h (Table 6). For the patients with a DWT > 7.5 h, their values of linear parameters improved. However, this improvement was not as significant as that of the angular parameters, where the values increased fivefold (SNB) and decreased fivefold (ANB and WITS).

**Table 6:** Comparison of the results in study and control groups Vergleich der Ergebnisse in Studien- und Kontrollgruppen

Variable	Study group (T1–T2)			Baccetti et al. [3]	Ghislanzoni et al. [9]	Cozza et al. [6]	Baysal and Uysal [4]
	DWT < 7.5 h	DWT > 7.5 h	Whole group				
Overjet	0.44	−2.87	−1.18	−0.12	0.1	−0.13	0.38
Co-Gn (mm)	3.97	6.14	5.03	–	4.9	3	3.83
Co-Go (mm)	2.43	2.84	2.63	1.25	3.1	–	1.98
Co-Olp	0.31	1.69	0.99	−0.20	–	0.8	0.75
Pg-Olp	2.6	5.22	3.89	0.90	–	2	2.12
WITS appraisal (mm)	0.24	−2.72	−1.21	–	0.3	–	–
SNA (°)	−0.07	−0.63	−0.35	–	0.2	0.33	–
SNB (°)	0.48	2.08	1.27	–	0.4	0.17	–
ANB (°)	−0.55	−2.73	−1.62	–	−0.3	0.13	–
Co-Go-Me (°)	−0.71	1.83	0.54	−1.32 (Ar-Go-Me)	−0.1	–	–

*DWT* daily wear time

After treatment, at T2, there was no patient with varying canine and molar relationships: Angle class II always coexisted with the canine class II, so did class I. Over half of the patients still demonstrated a full class II or cusp-to-cusp relationship. The Mann–Whitney U test revealed that the DWT was significantly (*p* < 0.05) longer in patients with established class I than in patients requiring further treatment (Table 7). The χ^2^ test demonstrated that achieving a class I relationship was achieved significantly more often in patients with a DWT > 7.5 h (*p* = 0.0009, Table 7).

**Table 7:** Statistical analysis of success and failure of class II treatment in relation to the DWT, overjet, age and gender Statistische Analyse von Erfolg bzw. Misserfolg der Klasse-II-Behandlung in Abhängigkeit von DWT, Overjet, Alter und Geschlecht

Canine and molar relationships at T2	DWT (h)					Overjet (mean values)		Age (mean values)		Gender	
	*n*	Mean	SD	< 7.5 h *n*/%	> 7.5 h *n*/%	T1	T2	T1	T2	Girls	Boys
I	24	8.97	1.97	5/17.8	19/70.3	8.33	3.67	10.9	12.4	13	11
II	31	6.54	3.45	23/82.2	8/29.6	8.08	9.59	10.8	12.39	16	15
*p*	–	0.00024		0.0009		–	–	–	–	–	–

*SD* standard deviation, *DWT* daily wear time

**Fig. 3:**
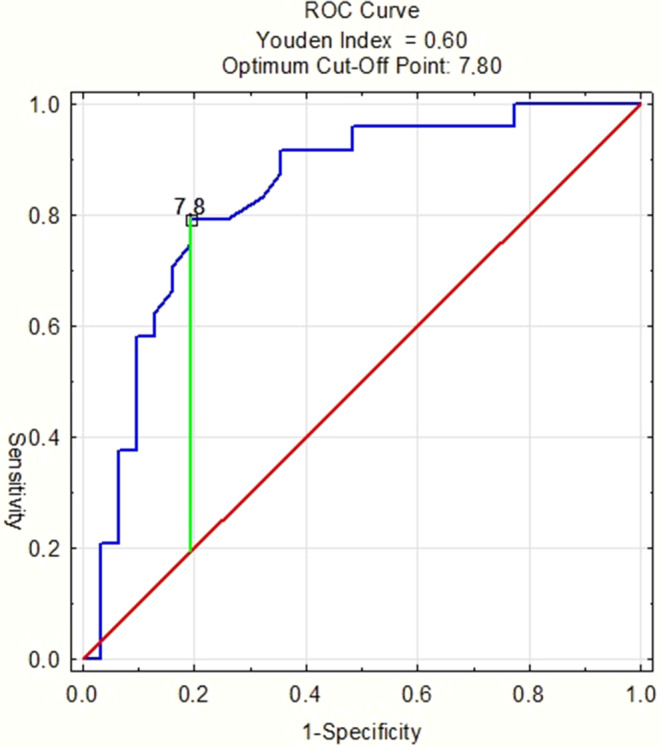
Receiver operating characteristic (ROC) curve. AUC = 0.835, SE = 0.056; *p* > 0.001 ROC(„receiver operating characteristic”)-Kurve. AUC = 0.835, SE = 0.056; *p* > 0.001

The calculated ROC curve (Fig. 3) made it possible to determine the minimum DWT required to establish a class I relationship. Since the area under the ROC curve equaled 0.835, it was determined that wearing the appliance for 7 h and 48 min a day provides an 83% probability of establishing a class I relationship.

## Discussion

Although the effectiveness of functional treatment of class II malocclusion has been often analyzed by researchers [5, 7, 10–13, 15–19, 21, 24, 29, 31], most of the study results have been inconsistent, mainly due to comparing fixed and removable functional appliances in terms of the treatment effects [4, 16, 18, 21]. This demonstrates that considering a control group is a major concern. Several authors overcame this problem [3, 4, 6, 9]. Ghislanzoni et al. [9] and Bacetti et al. [3] used data of untreated class II patients in their pubertal growth spurt extracted from the University of Michigan and Denver Child Growth Studies. In turn, Baysal and Uysal [4] and Cozza et al. [6] based their studies on skeletal maturity stages of class II patients aged 9–11 years, whose parents/guardians declined activator therapy. Such inclusion criteria were very similar to ours; therefore, we referred to the control groups of those authors. This approach is fully justified since exposing young patients to additional radiology exams seems to be ethically questionable in the absence of a decision on treatment.

The data of those patients who did not complete our study obviously could not be included in the analysis. However, it did not entitle us to assume patients’ poor compliance a priori, since they could either migrate or experience alteration of their treatment plan.

According to the meta-analysis by Ishaq et al. [11], dentoalveolar change is the main therapeutic effect of functional treatment, without pronounced effects on the skeleton, which is mainly determined by physiological growth. Livieratos and Johnston [13] also undermined the effects of functional therapy, stating that class II correction may be temporary due to transitional mandibular advancement. However, other authors proved that functional therapy makes it possible to achieve a permanent change in the mandibular growth direction and an increase in mandibular length [10, 16]. This is in accordance with our outcomes, especially with regard to sagittal changes. The increase in the SNB angle in patients with a DWT > 7.5 h was as high as 2.08°, which was a considerable improvement compared to both the control groups and patients with a DWT < 7.5 h. Similarly, Parekh et al. [22], who examined patients treated with the Twin Block appliance, observed an increase in the SNB angle by 1.47° and 1.54° for a DWT equaling 8.78 h and 12.38 h, respectively. According to Wieslander [30], such an increase in the SNB angle may be caused by several factors, including both natural mandibular growth and additional growth changes induced by functional therapy for class II malocclusion. The results of our study prove that it is, however, functional stimulation that leads to successful treatment of class II malocclusions since a DWT > 7.5 h significantly increased the SNB angle compared to a DWT < 7.5 h, with the final effect of reducing the ANB angle to 2.73° (Table 5).

Wieslander [30] reported that B‑point advancement could be attributed to a change in the position of the condylar within the temporomandibular joint; Johnston et al. [13] described these phenomena as a bodily functional shift. The results of our study provided evidence that the change in the position of the mandible is not merely the result of functional advancement but also the result of an increase in linear dimensions. We found that there is a considerable (r = 0.367) and significant (r = 0.569) relationship between increase in mandibular length, that is a Co-Gn distance increase, and a DWT > 7.5 h and a DWT < 7.5 h, respectively; this relationship confirms that improvements in the growth pattern of the mandible resulted from the functional treatment. Although increasing the DWT above the median only caused a tendency (*p* = 0.0569) for further improvement in the Co-Gn parameter, a DWT = 7.5 h must—in view of our results—be considered to be effective in terms of mandibular elongation. This observation also confirms the conclusion drawn by Parekh et al. [22].

We demonstrated a statistically significant (*p* < 0.05) improvement in the SNA parameter in patients adhering to a DWT > 7.5 h compared with patients with a DWT < 7.5 h. This finding may theoretically support the so-called “headgear effect” that has been described by several authors [19, 20, 29] as the outcome of functional treatment. Nevertheless, since the reduction equaled only 0.63°, we treat the statistical result with caution.

Regarding the vertical dimension of the mandible, our results proved that the values Co-Go significantly varied between patient adhering to DWTs < 7.5 h and DWTs > 7.5 h. Furthermore, a comparison of our results with the control groups from the studies by Ghislanzoni et al. [9] and Baysal and Uysal [4] revealed that DWTs > 7.5 h led to an increased vertical growth of ramus compared with the growth in untreated individuals. Franchi et al. [8] stated in their article that patients with Co-Go-Me angles smaller than 125.5° were more prone to functional treatment (better skeletal and dental effects can be expected). To exclude an influence of this parameter on the final outcome in our study, statistical analysis was performed (Table 8). No significant difference could be found between the group with a final class I relationship and the group with a class II relationship regarding the initial gonial angle. The same conclusion was drawn after analysis of the initial Co-Go-Me distribution in the group with DWTs longer and shorter than 7.5 h. Moreover, the mean values of gonial angles in each cohort were quite similar. All this information entitled us to claim that the Co-Go-Me angle was not a differentiating factor, which could pose a bias on our results. However, a significant correlation between the DWT and gonial angle change was observed (r = 0.85). Thus, a greater gonial angle increase can be expected in patients wearing Twin Block for more than 7.5 h daily.

**Table 8:** Statistical analysis of initial Co-Go-Me mean values in relation to the success or failure of class II treatment and DWT Statistische Analyse der Co-Go-Me-Mittelwerte zu Beginn in Bezug auf den Erfolg bzw. Misserfolg der Klasse-II-Behandlung und DWT

Canine and molar relationships at T2	Co-Go-Me (mean values in T1)	DWT	Co-Go-Me (mean values in T1)
I	125.08	< 7.5 h	125.31
II	125.48	> 7.5 h	125.29
*p*	> 0.05	*p*	> 0.05

*DWT* daily wear time

The aim of early functional treatment in a two-stage therapy is to facilitate later treatment with a fixed appliance [5]. If mechanical treatment starts in a patient with an Angle class I instead of II, this limits anchorage requirements, thus, facilitating this part of the therapy. In our study, 24 patients finished their treatment with good occlusal results, namely, reduced overjet, as well as molar and canine class I (Table 7). Thirty-one patients required further therapy with a fixed appliance, due to a full class II or cusp-to-cusp molar relationship. Initial overjet values in both groups were comparable. In Table 7, the distribution of treatment success regarding gender and age is also demonstrated. It can be clearly seen that neither gender nor age had an impact on good occlusal correction in the treated group. One patient, despite good compliance, ended his treatment with 14 mm of overjet. The etiology of this phenomenon can be either inherited or acquired. For example, juvenile idiopathic arthritis often causes a poor response to the functional stimuli provoked by the construction bite [23]. Our paper demonstrates that the mean DWT required to successfully treat class II malocclusion is 8.9 h. However, the comparative analysis of the DWT results provides evidence that a DWT > 7.5 h is already sufficient to correct the malocclusion since this improvement occurs statistically significantly more frequently than for patients adhering to DWT < 7.5 h. Moreover, analysis of the ROC curve revealed that the cut-off value, from which a statistically significant improvement in skeletal and occlusal parameters was observed, equaled 7.8 (approximately 8) hours. This is promising, especially because in our study, over half of the subjects did not comply with the recommended DWT of 10–14 h per day, which confirmed previously published results [25, 26]. Furthermore, we found that the DWT threshold value of 7.5 h, which, despite being almost half as low as the recommended DWT, makes it possible to treat class II malocclusions with a probability of more than 80% (Fig. 3). This is proven by a statistically significant correlation of the DWT with the changes of all measured parameters (Table 4), as well as by a significant improvement of the investigated cephalometric values in patients wearing the appliances for more than 7.5 h per day (Table 5). This is also demonstrated by the fact that for DWTs < 7.5 h, changes in angular measurements were comparable to those observed in untreated control groups from the studies by Ghislanzoni et al. [9] and Cozza et al. [6]. Finding evidence that the recommended DWT may be significantly shortened without compromising class II treatment efficiency is of major importance, as functional therapy with removable devices still has many advocates due to the reimbursement of such treatment costs from public funds.

## Conclusions

- The outcome of functional treatment of class II malocclusions with removable appliances depended on the daily wear time (DWT).
- The DWT threshold required to treat class II malocclusions using the Twin Block appliance was 8 h, which is promising in terms of achieving good patient compliance. In practice, the appliance may be worn only while sleeping.
- The efficient treatment of class II malocclusions within 18 months occurred significantly more often when the DWT was at least 7.5 h. In other words, the Twin Block appliance may be used as an inexpensive and effective therapeutic device, which may be paid for by public funds provided that the patient is properly qualified based on both his/her developmental age and cooperation.
- However, there were individuals who, regardless of their skeletal configuration being conducive to functional treatment and scrupulous adherence to the suggested DWT, did not respond to the therapy; this fact requires further investigation.
